# Differing Methods and Definitions Influence DALY estimates: Using Population-Based Data to Calculate the Burden of Convulsive Epilepsy in Rural South Africa

**DOI:** 10.1371/journal.pone.0145300

**Published:** 2015-12-23

**Authors:** Ryan G. Wagner, Fredrick Ibinda, Stephen Tollman, Lars Lindholm, Charles R. Newton, Melanie Y. Bertram

**Affiliations:** 1 Studies of the Epidemiology of Epilepsy in Demographic Surveillance Systems (SEEDS), INDEPTH Network, Accra, Ghana; 2 MRC/Wits Rural Public Health and Health Transitions Research Unit (Agincourt), School of Public Health, Faculty of Health Sciences, University of the Witwatersrand, Johannesburg, South Africa; 3 Epidemiology and Global Health, Department of Public Health & Clinical Medicine, Umeå University, Umeå, Sweden; 4 KEMRI/Wellcome Trust Research Programme, Centre for Geographic Medicine Research- Coast, Kilifi, Kenya; 5 Neurosciences Unit, Institute for Child Health, University College London, London, United Kingdom; 6 Department of Psychiatry, University of Oxford, Oxford, United Kingdom; 7 World Health Organization, Geneva, Switzerland; Université Catholique de Louvain, BELGIUM

## Abstract

**Background:**

The disability adjusted life year (DALY) is a composite measure of disease burden that includes both morbidity and mortality, and is relevant to conditions such as epilepsy that can limit productive functioning. The 2010 Global Burden of Disease (GBD) study introduced a number of new methods and definitions, including a prevalence-based approach and revised disability weights to calculate morbidity and new standard life expectancies to calculate premature mortality. We used these approaches, and local, population-based data, to estimate the burden of convulsive epilepsy in rural South Africa.

**Methods & Findings:**

Comprehensive prevalence, incidence and mortality data on convulsive epilepsy were collected within the Agincourt sub-district in rural northeastern South Africa between 2008 and 2012. We estimated DALYs using both prevalence- and incidence-based approaches for calculating years of life lived with disability. Additionally, we explored how changing the disease model by varying the disability weights influenced DALY estimates. Using the prevalence-based approach, convulsive epilepsy in Agincourt resulted in 332 DALYs (95% uncertainty interval (UI): 216–455) and 4.1 DALYs per 1,000 individuals (95%UI: 2.7–5.7) annually. Of this, 26% was due to morbidity while 74% was due to premature mortality. DALYs increased by 10% when using the incidence-based method. Varying the disability weight from 0.072 (treated epilepsy, seizure free) to 0.657 (severe epilepsy) caused years lived with disability to increase from 18 (95%UI: 16–19) to 161 (95%UI: 143–170).

**Conclusions:**

DALY estimates are influenced by both the methods applied and population parameters used in the calculation. Irrespective of method, a significant burden of epilepsy is due to premature mortality in rural South Africa, with a lower burden than rural Kenya. Researchers and national policymakers should carefully interrogate the methods and data used to calculate DALYs as this will influence policy priorities and resource allocation.

## Introduction

The Disability Adjusted Life Year (DALY) seeks to quantify the burden of disease in terms of both morbidity and mortality by combining years of life lost due to premature death (YLL) with years lived with disability (YLD) due to the disease [1,2]. In the 2010 Global Burden of Disease (GBD) study, the DALY relies on social preference and epidemiological data to determine disability weights (DW) and prevalence figures, respectively. Various theoretical and methodological challenges persist, which are likely to affect both the calculation and interpretation of DALY estimates.

Limited, reliable population-based data on disease parameters, specifically incidence and causes of death, make estimating the burden of a specific disease or condition difficult, especially in low- and middle- income countries (LMIC). The 2010 GBD study relies on a number of modeling techniques, including meta-regression and cause of death ensemble modeling [3], to estimate disease prevalence, incidence and duration and cause of death, respectively [4]. National and sub-national studies, using country-specific disease parameters would ensure that estimates are contextually accurate, thereby making them useful for justifying treatment options and performing cost-effectiveness analysis studies for national policy planning.

The 2010 GBD study, compared with earlier GBD studies, introduces a number of revised definitions and methods [4]. Included in these changes is a new standard life table that seeks to reflect the highest years of healthy life currently attainable globally (86.02 years at birth) equal for males and females. Additionally, DW for the 2010 GBD are derived from a global survey of more than 40,000 individuals, rather than from a panel of health care professionals as used in the past [5].

Previous GBD studies use the incidence-based approach for the calculation of YLD [4]. This method seeks to estimate YLD due to a condition by multiplying the incidence of the condition by the expected duration and DW. The 2010 GBD study calculates YLD using the prevalence-based approach, which estimates the current morbidity of a condition and also allows for the calculation of co-morbidities. Changing the approach will likely affect the results [6] given that prevalence is equal to the product of average incidence and average duration only when the age distribution of a population is static and the incidence and duration do not vary by age [7]. In this situation, incidence- and prevalence-based approaches would yield the same burden estimate. Using population-based prevalence and incidence figures, we examined the effect of these approaches on the burden of convulsive epilepsy, which only accounts for a fraction of all epilepsies, in rural South Africa.

Epilepsy is one of the most common, chronic, neurological disorders globally. The 2010 GBD study ranked idiopathic epilepsy, which is thought to account for 60–70% of all epilepsies [8] (and modeled to account for 58% in the 2010 GBD study [9]), 36^th^ in its contribution to the global burden, contributing more than 17.4 million DALYs and accounting for 0.75% of the global burden of disease [10]. Of this burden, 80% is estimated to be in LMIC [11] where epilepsy remains largely untreated, especially in rural areas [12] where the prevalence and incidence is often highest [13,14].

Morbidity accounts for half of the global burden of epilepsy, while premature mortality accounts for the other half [9]. The DW associated with severe epilepsy in the 2010 GBD study ranks amongst the highest of any condition or illness [5], potentially contributing, along with updated data, methods and definitions, to higher 2010 burden estimates when compared with earlier figures [10]. Epilepsy ranks 19^th^ in its contribution to the total disease burden in both southern and eastern sub-Saharan Africa and 14^th^ in western sub-Saharan Africa—higher than anywhere else in the world [10]. Yet these figures are regional, resulting in uncertainty as to the generalizability of these findings to a national or sub-national level, making national level policy planning difficult [15].

Few LMIC national or sub-national studies explore the burden of epilepsy and none from South Africa, partially due to paucity of disease-specific data that are required to calculate DALYs [15]. Having measured the prevalence, incidence and mortality of convulsive epilepsy in rural South Africa between 2008 and 2012 [16,17], we estimated the burden of convulsive epilepsy in terms of DALYs. Using both the prevalence- and incidence-based approach and varying the DW in the disease model, we explore the effects of these variations on the calculated DALYs attributable to convulsive epilepsy in a rural South African population.

## Methodology

### Research setting

The Agincourt sub-district, which is covered by a well-established health and socio-demographic surveillance system (HDSS) established in 1992, lies 500 kilometers northeast of Johannesburg in the South African province of Mpumalanga (http://www.agincourt.co.za). Constituting the southern half of the Bushbuckridge sub-district, the Agincourt research site consists of 420 km^2^ of semi-arid scrubland.

#### Study population

Trained fieldworkers annually visit each household within the study site to update vital events, including births, deaths and migrations [18]. In 2008, the Agincourt HDSS population was comprised of 83,121 individuals in 25 research villages.

#### Measures of morbidity & mortality

The HDSS has also served as a platform to screen for individuals who potentially have a specific condition in order to estimate prevalence levels [16]. In 2008, a three-stage, population-based study, coupled with a clinical history-taking and assessment by a trained nurse and confirmed by the study neurologist (CRN), identified people with active convulsive epilepsy (CE), resulting in an adjusted prevalence of 7.0 per 1,000 individuals [16]. The methods used in this study have been previously discussed elsewhere [16,19]. The population was subsequently followed up for a four-year period to determine the mortality due to CE [17].

A follow-up, cross-sectional study in 2012 across the whole of Agincourt, used the same methodology as the 2008 prevalence study to establish the number of incident CE cases amongst those who had been found not to have epilepsy in 2008 [17]. All individuals who formed part of the 2008 survey and were not found in the 2012 survey due to migration were coded as ‘lost to follow-up’ and excluded from analysis [17].

### Analysis

All data were entered into a mySQL database (OracleCorp, Redwood Shores, CA, U.S.A.) and analyzed using Stata 13 (College Station, TX, U.S.A.). Prevalence, incidence and standardized mortality ratios (SMR) for CE were calculated, by sex, and 6 age bands used in previous epilepsy studies from Africa [16,19,20]: 0–5, 6–12, 13–18, 19–28, 29–49 and 50+ years. These parameters were then entered into DisMod II (http://www.who.int/healthinfo/global_burden_disease/tools_software/en/) a software program that models epidemiological measures of a disease when only a limited number of disease parameters exist [21]. DisMod II evaluates the internal consistency of the input parameters and, using a set of differential equations, models epidemiological parameters by relying on the inter-relationship between the input parameters [21]. The 2008 population age structure and the Agincourt mortality rates (Table 1 *)*, which were calculated by dividing the number of deaths in Agincourt HDSS between 2008–2012 by the number of person-years observed, by sex, in each age band, were also entered into DisMod II.

**Table 1:** Age- and sex-specific demographics and mortality rates of Agincourt HDSS, 2008–2012.

	Population				All-Cause Mortality 2008–12 (per 1,000 pyo*)			
Age band (in years)	Male pyo*	Male deaths	Female pyo*	Female deaths	Male (95%CI^^^)		Female (95%CI^^^)	
***0–5***	20675	80	20539	52	3.9	(3.1–4.8)	2.5	(1.9–3.3)
***6–12***	23729	31	24334	28	1.3	(0.9–1.9)	1.2	(0.8–1.7)
***13–18***	19126	17	18385	17	0.9	(0.6–1.4)	0.9	(0.6–1.5)
***19–28***	37381	163	34081	191	4.4	(3.7–5.1)	5.6	(4.9–6.5)
***29–49***	31692	592	35511	424	18.7	(17.2–20.2)	11.9	(10.9–13.1)
***50+***	13401	584	21773	638	43.6	(40.2–47.3)	29.3	(27.1–31.7)
***All Ages***	146004	1467	154623	1350	10.0	(9.5–10.6)	8.7	(8.3–9.2)

*pyo: Person-years observed

^^^CI: confidence interval

The output measures from DisMod II included incidence, prevalence, remission, duration of epilepsy and SMR, with the quality of the output measures directly dependent on the quality of the input parameters [22]. The results, smoothed using inbuilt functions—piecewise linear interpolation, moving average and cubic spline- were presented in 6 age bands for both males and females (Table 2) with 95% uncertainty intervals (95%UI). To generate the 95%UI, the input parameters were assumed to be normally distributed. The 95%UI were generated using Monte Carlo simulation with 1,000 iterations.

**Table 2:** DisMod II output for males and females- age-specific incidence, prevalence, remission, duration and standardized mortality rates, Agincourt 2008–2012.

Age band (in years)	Incidence per 100,000 (95%UI)	Prevalence per 1,000 (95%UI)	Remission per 100 (95%UI)	Duration in years (95%UI)	Standardized Mortality Rate (95%UI)
**Male**					
***0–5***	30.1 (27.7–32.6)	1.4 (1.2–1.6)	16.0 (14.8–17.1)	12.0 (11.3–12.7)	7.7 (7.3–8.1)
***6–12***	23.6 (21.8–25.4)	1.9 (1.6–2.2)	3.3 (3.1–3.6)	18.4 (17.3–19.4)	20.2 (19.3–21.2)
***13–18***	15.0 (13.6–16.4)	2.6 (2.3–2.9)	4.5 (4.2–4.8)	16.5 (15.5–17.5)	11.5 (11.0–12.2)
***19–28***	15.0 (13.8–16.2)	1.9 (1.6–2.3)	9.3 (8.8–9.8)	26.0 (24.6–27.4)	1.9 (1.8–2.0)
***29–49***	16.9 (15.7–18.2)	3.3 (3.0–3.6)	0.0 (0.0–0.1)	32.0 (30.1–33.8)	2.0 (1.9–2.1)
***50+***	19.7 (18.3–21.2)	5.6 (5.1–6.0)	2.0 (1.6–2.4)	25.3 (22.3–28.5)	3.6 (3.4–3.7)
***All ages***	19.4 (17.9–21.0)	2.6 (2.3–2.9)	4.6 (4.1–5.0)	21.5 (20.1–22.8)	8.2 (7.9–8.7)
**Female**					
***0–5***	16.0 (13.1–18.0)	0.4 (0.3–0.5)	30.5 (28.5–32.6)	10.6 (10.11.3)	1.0 (1.0–1.0)
***6–12***	19.3 (17.2–20.2)	0.9 (0.7–1.0)	6.6 (5.6–7.3)	28.2 (26.7–29.6)	1.0 (1.0–1.0)
***13–18***	17.8 (16.2–18.8)	1.9 (1.7–2.1)	0.4 (0.2–0.6)	27.2 (25.8–28.7)	3.7 (3.6–4.0)
***19–28***	13.0 (11.9–14.1)	2.8 (2.5–3.0)	1.3 (0.9–1.7)	22.4 (21.3–23.6)	2.7 (2.8–2.9)
***29–49***	16.1 (15.0–17.4)	3.9 (3.5–4.2)	3.1 (2.8–3.4)	16.6 (15.7–17.5)	1.3 (1.1–1.4)
***50+***	24.2 (22.4–26.0)	3.6 (3.3–3.9)	6.5 (6.0–7.0)	14.2 (13.1–15.3)	1.7 (1.6–1.7)
***All ages***	17.2 (15.5–18.5)	2.4 (2.2–2.7)	3.9 (3.4–4.5)	19.7 (18.7–20.7)	2.6 (2.5–2.7)

#### Disability weights

The 2010 GBD reported differing DW related to differing disease states marked by whether or not an individual was on treatment and frequency of seizures [5]. These, in turn, were derived from a survey of more than 40,000 respondents from a number of countries and settings [5]. The DW used for YLD calculations was 0.346, which is the mean DW for epilepsy used in GBD 2010 for the sub-Saharan African region (Theo Vos, personal communication) and has been used elsewhere [20].

Life expectancy values used to calculate DALYs were those used in the 2010 GBD study, with a life expectancy of 86.02 years at birth for both males and females [23]. Age weighting and discount rates (social weighting) were not used in this study, in line with the 2010 GBD study [23].

#### DALY calculation using prevalence-based approach

DALYs, the sum of YLD and YLL, were calculated first using the prevalence-based approach, which was employed in the 2010 GBD study. For this approach, the prevalence of CE, for each age band, was multiplied by the DW to yield YLD:

$${YLD}_{p}=\;\text{Prevalenc}e\;\times \;\text{DW}$$ (1)

YLL were calculated as the number of deaths (directly attributable to epilepsy) per year multiplied by the standard life expectancy at the age of death in both the prevalence- and incidence-based approaches.

DALYs are presented as the sum of YLL and YLD and reported both as absolute and relative numbers (per 1,000 individuals). The results are presented by both age band and sex.

#### DALY calculation using incidence-based approach

In order to determine the differences between the prevalence- and incidence-based approach used in previous versions of the GBD, we also calculated YLD, for both males and females, using the formula incidence (per year) multiplied by DW and average duration (in years) to epilepsy remission or death [24].

$${YLD}_{i}=\;\text{Incidence}\;\times \;\text{Duration}\;\times \;\text{DW}$$ (2)

To allow for comparison, the prevalence, incidence and duration used in both approaches were the output parameters generated by DisMod II to allow for internal consistency. We assumed that no significant change in the incidence of convulsive epilepsy occurred between 2008 and 2012 and hence, we used 2008 as the base year for calculating YLDs using the incidence-based approach. This allowed for comparison with the prevalence-based approach to calculate YLDs, which relied on 2008 prevalence figures. We further present absolute YLD calculations using the incidence-based approach in S2 Table.

#### Sensitivity analysis

All YLL, YLD and DALY calculations are presented with 95% bootstrapped uncertainty intervals (95%UI) calculated using the R boot package (with 1,000 iterations) which is implemented in R, an open-source statistical software [25–27]. The uncertainty intervals take into account the uncertainty in the epidemiological parameters used to estimate YLL, YLD and DALY.

In order to explore the effect on DALYs of changing the disease model by varying DW, one-way sensitivity analyses were undertaken. DW of epilepsy-related health states have been shown to vary based on seizure type and seizure frequency and are related to functional status and quality of life at each state [28]. We used each of the 4 DW from the GBD 2010 study- representing varying degrees of epilepsy severity- to examine the sensitivity of DW on YLD. The four DWs used were: 0.072 (treated, seizure free), 0.319 (treated with recent seizures), 0.42 (untreated epilepsy) and 0.657 (severe epilepsy). We included treated, seizure free as a possible health state as the definition of convulsive epilepsy used in this study included patients with a history of convulsive epilepsy whose seizures were controlled using anti-epileptic drugs [16].

#### Ethical considerations

The data used in this study were collected as part of the Studies of the Epidemiology of Epilepsy in Demographic Surveillance Systems (SEEDS) study by a team of researchers within the MRC/Wits Agincourt Research Unit. The SEEDS study received ethical approval from the University of the Witwatersrand Human Research Ethics Committee (M080455, M120660) and the Mpumalanga Province Department of Health's Research and Ethics Committee. No additional data were collected for the purposes of this study and the authors did not have access to any identifying information for this study. The data were de-identified previously by the Agincourt researchers in the SEEDS study. All of the data on which this work is based are provided in the paper and supporting information tables.

## Results

Convulsive epilepsy was found to be responsible for 332 DALYs (95%UI: 216–455) in Agincourt using the prevalence-based approach, the GBD life table and the mean DW (0.346) for epilepsy in sub-Saharan Africa (Table 3). These figures equated to 4.1 (95%UI: 2.7–5.7) DALYs per 1,000 individuals per annum (Table 4). Eighty-five DALYs (95%UI: 77–94) were due to YLD (26% of total DALYs), while 247 DALYs (95%UI: 129–373) were due to YLL (74%) due to CE (Table 3).

**Table 3:** Absolute Disability-adjusted life years (DALYs) calculated using prevalence method by age and sex, Agincourt 2008–2012.

Age band	YLL (95%UI)	YLD (95%UI)	DALYs (95%UI)
**Male**			
***0–5***	0 (0–0)	2.8 (1.4–4.5)	2.8 (1.4–4.5)
***6–12***	58.4 (0–118.8)	6.6 (4.2–9.3)	64.9 (5.2–151.3)
***13–18***	0 (0–0)	6.9 (4.5–9.3)	6.9 (4.5–9.3)
***19–28***	0 (0–0)	6.6 (4.2–9.3)	6.6 (4.2–9.3)
***29–49***	38.2 (0–93.5)	15.6 (11.8–19.4)	53.7 (13.8–95.7)
***50+***	55.1 (14.1–101.8)	6.2 (4.1–8.6)	61.4 (19.3–112.5)
***All ages***	151.6 (66.3–249.1)	44.6 (38.4–50.5)	196.3 (109.8–295.5)
**Female**			
***0–5***	0 (0–0)	0.7 (0–1.7)	0.7 (0–1.7)
***6–12***	0 (0–0)	3.5 (1.7–5.5)	3.5 (1.7–5.5)
***13–18***	0 (0–0)	4.8 (2.8–7.3)	4.8 (2.8–7.3)
***19–28***	47.8 (0–118.2)	9.7 (6.9–12.8)	57.4 (8.3–110.6)
***29–49***	21.7 (0–65.2)	14.5 (11.1–18.3)	36.3 (11.8–79.4)
***50+***	26.2 (0–63.9)	6.9 (4.5–9.4)	33.2 (5.5–69.7)
***All ages***	95.7 (33.0–168.6)	40.1 (33.9–46.4)	135.9 (66.9–210.2)
**Both male and female**			
***0–5***	0 (0–0)	3.5 (1.7–5.2)	3.5 (1.7–5.2)
***6–12***	58.4 (0–145.7)	10.0 (7.3–13.2)	68.4 (8.6–155.4)
***13–18***	0 (0–0)	11.8 (8.6–15.2)	11.8 (8.6–15.2)
***19–28***	47.8 (0–118.2)	16.3 (12.5–20.1)	64.0 (14.5–130.5)
***29–49***	59.9 (18.42–118.4)	30.1 (24.9–35.3)	90.0 (33.5–151.7)
***50+***	81.4 (26.88–137.2)	13.2 (10.0–16.6)	94.5 (40.0–155.1)
***All ages***	247.4 (129.4–373.2)	84.8 (76.5–93.8)	332.1 (215.9–454.8)

**Table 4:** Relative Disability-adjusted life years (DALYs) calculated using prevalence-method by age and sex per 1,000 individuals, Agincourt 2008–2012.

Age band	YLL per 1,000 (95%UI)	YLD per 1,000 (95%UI)	DALYs per 1,000 (95%UI)
**Male**			
***0–5***	0 (0–0)	0.5 (0.2–0.8)	0.5 (0.2–0.8)
***6–12***	9.2 (0–18.5)	1.0 (0.6–1.5)	10.2 (0.8–23.9)
***13–18***	0 (0–0)	1.1 (0.7–1.5)	1.1 (0.7–1.5)
***19–28***	0 (0–0)	0.8 (0.5–1.0)	0.8 (0.5–1.0)
***29–49***	4.4 (0–9.2)	1.8 (1.4–2.2)	6.2 (1.6–12.6)
***50+***	14.8 (3.8–27.1)	1.7 (1.0–2.3)	16.5 (5.2–28.9)
***All ages***	3.9 (1.7–6.2)	1.1(1.0–1.3)	5.0 (2.8–7.4)
**Female**			
***0–5***	0 (0–0)	0.1 (0–0.3)	0.1 (0–0.3)
***6–12***	0 (0–0)	0.5 (0.3–0.8)	0.5 (0.3–0.8)
***13–18***	0 (0–0)	0.8 (0.5–1.1)	0.8 (0.5–1.1)
***19–28***	5.5 (0–11.6)	1.1 (0.8–1.5)	6.6 (1.0–14.7)
***29–49***	2.3 (0–6.8)	1.5 (1.2–1.9)	3.8 (1.2–8.4)
***50+***	4.5 (0–9.5)	1.2 (0.8–1.6)	5.7 (1.0–12.3)
***All ages***	2.3 (0.6–4.0)	1.0 (0.8–1.1)	3.2 (1.5–5.0)
**Both male and female**			
***0–5***	0 (0–0)	0.3 (0.2–0.5)	0.3 (0.2–0.5)
***6–12***	4.5 (0–9.2)	0.8 (0.6–1.0)	5.3 (0.7–11.9)
***13–18***	0 (0–0)	1.0 (0.7–1.2)	1.0 (0.7–1.2)
***19–28***	2.7 (0–6.8)	0.9 (0.7–1.2)	3.7 (0.9–6.8)
***29–49***	3.3 (1.0–6.8)	1.6 (1.4–1.9)	4.9 (1.9–8.4)
***50+***	8.5 (3.0–15.1)	1.4 (1.1–1.8)	9.9 (4.1–16.0)
***All ages***	3.0 (1.6–4.5)	1.0 (0.9–1.1)	4.1 (2.7–5.7)

Males were found to have a higher burden, in terms of DALYs than females, with males contributing 196 (59% of the total number of DALYs) DALYs and females contributing 136 DALYs (41% of total) (Table 3). Males with CE had a burden of 5.0 (95%UI: 2.8–7.4) DALYs per 1,000 individuals, while females had a burden of 3.2 (95%UI: 1.5–5.0) DALYs per 1,000 individuals (Table 4).

### Prevalence- versus incidence-based YLD calculations

The prevalence-based approach to calculating YLD resulted in 38% and 29% less YLD for males and females, respectively, compared to incidence-based approach (Table 5), with males contributing 56.7 YLD (48.9–65.1) per annum and females 49.8 (42.6–56.2) per annum (S2 Table). This corresponded to 10% less DALYs overall, using the prevalence-based YLD calculations for both males and females.

**Table 5:** Comparison of results using Incidence- versus Prevalence-based method of calculating DALYs.

Method	Population	Incidence per 100,000	Prevalence per 1,000	Duration	Disability Weight	YLD	Δ% of YLD	Δ% of DALYs
**Male**								
***Incidence***	39313	19.4	.	21.5	0.346	56.7	**38%**	**10%**
***Prevalence***	39313	.	2.6	.	0.346	35.4		
**Female**								
***Incidence***	42443	17.2	.	19.7	0.346	49.8	**29%**	**10%**
***Prevalence***	42443	.	2.4	.	0.346	35.2		

#### Sensitivity analysis

A two-fold increase in YLD (Table 6) and 23% more overall DALYs were observed when the disease model was changed to reflect the DW for severe epilepsy.

**Table 6:** YLD estimates using varying disability weights, Agincourt 2008–12.

	GBD 2010 DW for SSA region, 0.346 (95%UI*)	GBD 2010 DW treated, seizure free, 0.072 (95%UI*)	GBD 2010 DW treated with recent seizures, 0.319 (95%UI*)	GBD 2010 DW untreated epilepsy, 0.42 (95%UI*)	GBD 2010 DW for severe epilepsy, 0.657 (95%UI*)
***Male***	44.6 (38.4–50.5)	9.3 (7.6–9.6)	41.2 (37.7–47.8)	54.2 (44.4–59.9)	84.8 (74.0–98.6)
***Female***	40.1 (33.9–46.4)	8.4 (6.9–8.9)	37.0 (28.7–39.5)	48.7 (45.3–54.5)	76.2 (64.6–83.8)
***Both sexes***	84.8 (76.5–93.8)	17.6 (16.4–18.6)	78.2 (75.6–82.6)	102.9 (92.1–112.5)	161.0 (142.9–170.5)

* Uncertainty Intervals calculated using bootstrapping with 1,000 iterations

## Discussion

### Prevalence- versus incidence-based YLD calculations

Overall DALY estimates were 10% lower when using the prevalence-based method versus the incidence-based method to calculate YLD. The lower figure was not an actual reduction in the burden of CE, but rather different measures of the burden of CE. In situations where the age-structure and size of the population as well as the incidence and duration of the disease do not vary by age, using the prevalence-based approach will yield comparable estimates. The 10% difference between the two approaches in this study could be due to incidence rates that reflect the changing population age structure of Agincourt, with increases in both the very young and the old [18], both groups with high epilepsy incidence [17].

An incidence-based approach to calculating YLDs is likely more responsive to demographic transitions, especially when the condition under investigation varies by age. For cost-effectiveness analyses of interventions, using the incidence-based approach would likely be preferred, as it captures the change in future gains. However, in many LMIC settings, the paucity of incidence data makes incidence-based DALY estimates difficult. Nevertheless, use of the same methodology is imperative when undertaking comparative burden analyses.

We found convulsive epilepsy to account for 4.1 (95%UI: 2.7–5.7) DALYs per 1,000 individuals per annum in rural, northeast South Africa using the prevalence-based approach and average DW for sub-Saharan Africa (0.346) used in the 2010 GBD study. Our results are less than the 5.3 (95%UI: 4.2–7.6) DALYs per 1,000 individuals per annum for South Africa reported in the 2010 GBD study, which explored the burden of all idiopathic epilepsies and not just convulsive epilepsy [10]. While convulsive epilepsies are likely to contribute the majority of the burden due to their significant disability, non-convulsive epilepsies, which make up 50% of all epilepsies [29], are likely to contribute modestly to the overall burden of epilepsy, since they are associated with less severity (reflected in the DW) and mortality.

Our DALY point estimates were lower than the 4.3 (95%UI: 3.4–5.2) DALYs per 1,000 individuals found in rural, coastal Kenya [20]. The difference is likely due to the higher prevalence, incidence and mortality due to CE found in the Kenyan population [20], which in turn is likely due to a greater prevalence of known parasitic and perinatal risk factors [19,30].

Twenty-six percent (84.8 (95%UI: 76.5–93.8)) of DALYs were attributable to disability while 74% (247.4 (95%UI: 129.4–373.2) were attributable to early mortality in rural South Africa. This finding highlights the fact that while epilepsy is a chronic condition, it is associated with substantial mortality, as seen in the modeled SMRs (8.2 (95%UI: 7.9–8.7) for males and 2.6 (95%UI: 2.5–2.7) for females) and also reported previously. The composition of the burden, in terms of YLL and YLD, was similar to that found in rural Kenya [20]. However, based on the DisMod II estimates, the duration of CE in South Africa was significantly longer for both males (21.5 years (95%UI: 20.1–22.8) versus 8.56 years (95UI: 2.84–13.56)) and females (19.7 years (95%UI: 18.7–20.7) versus 7.98 years (95%UI: 3.29–13.06)). The shorter duration found in Kenya is likely due to significantly higher mortality rates- especially amongst females found in rural Kenya [20]. The higher risk of dying likely results in a shorter duration of epilepsy.

### Sensitivity analysis

One-way sensitivity analysis suggests that changing disease model by varying the DW of epilepsy can substantially affect DALYs due to epilepsy, which has also been shown in other studies [31,32]. The 2010 GBD no longer relies on expert opinion to determine DW, but instead bases DW on both in-person interviews with nearly 14,000 individuals from high-, middle- and low-income countries as well as an online survey [5]. The resultant DW for epilepsy are substantially higher than those used in previous GBD studies. Severe epilepsy now ranks amongst the most disabling conditions—higher than AIDS without anti-retroviral therapy, terminal cancer, or severe stroke with long-term consequences, including cognition problems [5]. Using GBD 2010 DW estimates for treated, seizure free epilepsy reduced overall DALYs by 20%. A 20% reduction in the burden of epilepsy is equivalent to averting 3,241 DALYs per a one million-person population. Alternatively, when using the DW for severe epilepsy, a two-fold increase in YLD and a 23% increase in overall DALYs resulted.

Epilepsy can cause significant disability and early mortality, especially in LMIC. Using global (or regional) DW for epilepsy allows for comparison across studies, but it also may influence national or sub-national burden estimates by misappropriating the actual disability or early mortality associated with epilepsy in that context. As such, efforts should be made to develop contextually appropriate tools to accurately quantify the disability due to epilepsy [33] when undertaking national or sub-national disease burden analyses to assist with national policy planning or resource allocation.

The DALY seeks to capture the disability and premature death associated with a given condition. However, the DALY does not account for the ‘social disability’ that results from a given condition, which can be sizable. Epilepsy is associated with substantial stigma, which has been shown to vary by context [11]. In some parts of sub-Saharan Africa, having epilepsy results in reduced marriage prospects, less access to education and lower employment opportunities [34]. This places an additional burden on the individual with epilepsy. Including a ‘social disability weight’ when calculating YLD may allow for a more holistic, contextual representation of the total burden of a condition and could be useful for allocating resources within that context.

### Limitations

Overall, this study provides a robust estimate of the burden of convulsive epilepsy in rural, northeast South Africa due to the use of reliable data. Yet this study likely underestimates the total burden of epilepsy as result of only exploring convulsive epilepsy, which is estimated to be only 50% of the total number of cases of epilepsy [29]. While convulsive epilepsy has been found to be associated with the greatest proportion of morbidity (stigma, burns, etc.) and mortality [35], a modest proportion of the total burden attributable to epilepsy may be missed by only exploring convulsive epilepsy.

Studies have shown that the prevalence [13], incidence [14] and mortality [17,36] of epilepsy in rural areas of LMIC are higher than in urban areas. As such, this study, which relies on rural epidemiological parameters of convulsive epilepsy, is limited in its generalizability. Urban areas, experiencing lower prevalence and incidence, have also been found to have lower treatment gap figures [12], which could also affect burden estimates. It is possible that the burden of epilepsy in rural South Africa will differ from the burden in urban South Africa.

Finally, the use of the DALY is not without critique [37] and a number of potential challenges have been highlighted in this study, including the effects of selection of the correct disease model and corresponding DW, which could result in inaccurate reporting of national disease burden in LMIC [38]. It is essential to recognize what DALY estimates represent, especially for those using DALYs to assist with national policymaking and resource allocation in LMIC. It is plausible, and has been shown in at least one study, that general health state preferences and subsequent DW can differ among different cultures [39]. Some critics argue that by DALYs failing to correctly measure disability [40,41] individuals with disability are undervalued [37,42]. However, even in light of these shortcomings, the DALY provides a useful, metric to measure, and using the same methodology, compare the burden of a given condition or disease- including epilepsy.

## Conclusion

Convulsive epilepsy causes a substantial burden in rural South Africa, though point estimates are lower than that reported in rural Kenya and the 2010 GBD study. Differing disease profiles and type of epilepsies explored are likely responsible for the observed differences. Varying methodologies used to calculate DALYs and disability weights affected the estimated burden by as much as 24%. Researchers and national policymakers should take care when interpreting DALY estimates. Efforts aimed at exploring valuation of disability in rural South African could assist in ascertaining a more accurate estimate of the burden of convulsive epilepsy.

## Supporting Information

S1 Table DisMod II input file with Agincourt epidemiological parameters. (DOCX)

S2 Table Absolute YLDs, by sex and age band, derived using the incidence-based method for calculating YLDs. (DOCX)
