# Supplementary material for: Differing Methods and Definitions Influence DALY estimates: Using Population-Based Data to Calculate the Burden of Convulsive Epilepsy in Rural South Africa
Source: PLoS One. 2015 Dec 23;10(12):e0145300. doi: 10.1371/journal.pone.0145300 (PMC4689490; doi:10.1371/journal.pone.0145300)
Supplement: S1 Table — (DOCX) [file pone.0145300.s001.docx]

| **S2 Table**. DisMod II input file with Agincourt epidemiological parameters. | | | |
| --- | --- | --- | --- |
| **Males** | | | |
| Age | Incidence (rates) | Prevalence (rates) | RR Mortality (rates) |
| 0-5 | 0.0003 | 0.0014 | 7.1 |
| 6-12 | 0.0002 | 0.003 | 29.3 |
| 13-18 | 0.0001 | 0.004 | 0 |
| 19-28 | 0.0002 | 0.0019 | 2.23 |
| 29-49 | 0.0002 | 0.0049 | 2.05 |
| 50+ | 0.0002 | 0.0056 | 3.364 |
| **Females** | | | |
| Age | Incidence (rates) | Prevalence (rates) | RR Mortality (rates) |
| 0-5 | 0.0002 | 0.0004 | 0 |
| 6-12 | 0.0002 | 0.0015 | 0 |
| 13-18 | 0.0002 | 0.0028 | 19.66 |
| 19-28 | 0.0001 | 0.0029 | 4.57 |
| 29-49 | 0.0002 | 0.0043 | 0.94 |
| 50+ | 0.0002 | 0.0036 | 1.67 |
