# Supplementary material for: Differing Methods and Definitions Influence DALY estimates: Using Population-Based Data to Calculate the Burden of Convulsive Epilepsy in Rural South Africa
Source: PLoS One. 2015 Dec 23;10(12):e0145300. doi: 10.1371/journal.pone.0145300 (PMC4689490; doi:10.1371/journal.pone.0145300)
Supplement: S2 Table — (DOCX) [file pone.0145300.s002.docx]

| **Supplementary Table 2:** Absolute YLDs, presented by age band and sex, derived using the incidence-based method for calculating YLDs | |
| --- | --- |
|  |  |
| **Age band (in years)** | **YLD (95%UI)** |
| **Male** | |
| ***0-5*** | 7.1 (6.2-8.2) |
| ***6-12*** | 9.5 (8.3-10.8) |
| ***13-18*** | 4.3 (3.7-5.0) |
| ***19-28*** | 13.4 (11.6-15.2) |
| ***29-49*** | 16.1 (14.1-18.3) |
| ***50+*** | 6.4 (5.3-7.8) |
| ***All ages*** | 56.7 (48.9-65.1) |
| **Female** | |
| ***0-5*** | 3.3 (2.6-4.0) |
| ***6-12*** | 12.3 (10.4-13.6) |
| ***13-18*** | 8.4 (7.2-9.3) |
| ***19-28*** | 9.8 (8.5-11.2) |
| ***29-49*** | 8.9 (7.9-10.2) |
| ***50+*** | 7.0 (5.9-8.1) |
| ***All ages*** | 49.8 (42.6-56.2) |
